# Type 1 insulin-like growth factor receptor targeted therapies in pediatric cancer

**DOI:** 10.3389/fonc.2013.00009

**Published:** 2013-02-04

**Authors:** Michael J. Wagner, Robert G. Maki

**Affiliations:** ^1^Department of Medicine, Mount Sinai Medical CenterNew York, NY, USA; ^2^Department of Medicine, Mount Sinai School of MedicineNew York, NY, USA; ^3^Department of Pediatrics, Mount Sinai School of MedicineNew York, NY, USA; ^4^Department of Orthopedics, Mount Sinai School of MedicineNew York, NY, USA

**Keywords:** insulin-like growth factor pathway, pediatric cancer, sarcoma, resistance mechanisms, IGF-1R

## Abstract

Data from over 20 years ago demonstrated potential use for insulin-like growth factor (IGF) signaling modulators, specifically with IGF-1R antagonists, in a variety of pediatric and adolescent cancers, particularly in sarcomas. However, in spite of promising preclinical data, IGF-1R inhibitors have not had the success as single agents that was originally hoped for in clinical trials. Several potential mechanisms exist by which tumors are resistant to IGF-1R inhibitors. Notably, these resistance mechanisms are currently best understood in Ewing sarcoma and alveolar rhabdomyosarcoma. Various treatment schema have been proposed as a potential way to overcome this resistance. The use of IGF-1R inhibitors, mechanisms of resistance, and current ongoing clinical studies using IGF-1R inhibitors in pediatric cancers are reviewed here.

## INTRODUCTION

The insulin-like growth factor (IGF) pathway regulates cellular growth, proliferation, and survival, and is important in the development of several cancers, including pediatric sarcomas, gliomas, neuroblastoma, and medulloblastoma. There are three main ligands that activate the IGF signaling cascade: IGF1, IGF2, and insulin. These ligands are bound to one of six binding proteins in circulation, IGFBP1–6. The IGF binding proteins, in particular IGFBP3, are implicated in modulating interactions between ligand and receptor. Some have negative effects on receptor signaling by competing for ligand. Others, notably IGFBP2 and IGFBP5, amplify IGF signaling (reviewed in Grimberg and Cohen, 2000). Higher levels of extracellular IGFBP3 are thought to decrease signaling via IGF-1R even independent of ligand (Ricort and Binoux, 2001). IGF binding proteins are also found intracellularly. The IGF binding proteins have a wide range of functions and have been implicated in signaling cascades other than IGF signaling (reviewed in Grimberg and Cohen, 2000; Weroha and Haluska, 2012). Further research is needed to elucidate their many biological roles and their precise role in cancer development.

There are two IGF receptors, IGF-1R and IGF-2R, with IGF-1R as the more important positive regulator of the IGF initiated cascade as it is the only one with an intracellular kinase domain (De Meyts and Whittaker, 2002). In contrast to the activating effects of IGF-1R signaling, IGF-2R is thought to be a negative regulator of IGF signaling by binding extracellular IGF2 (Ghosh et al., 2003). Unlike most other receptor tyrosine kinases, IGF-1R and insulin receptors exist as dimers prior to ligand binding. These receptors can act as either homodimers or a variety of heterodimers. Either variation can take part in tumorigenesis.

Binding of ligand to IGF-1R leads to autophosphorylation of the intracellular domain and recruitment of adapter proteins, ultimately leading to activation of a signaling cascade that can include modulation of PI-3-kinase/AKT/mTOR and Ras/Raf/MAPK, leading to cell proliferation (reviewed in Pollack, 2008; Rikhof et al., 2009; Maki, 2010).

In spite of very encouraging preclinical results, initial clinical studies of IGF-1R antagonists as a single agent have not demonstrated the results that were initially anticipated in clinical trials. Several potential mechanisms exist by which tumors are resistant to IGF-1R inhibitors. Various treatment schema have been proposed as a potential way to overcome this resistance. The preclinical data in pediatric cancers, mechanisms of resistance, and current ongoing clinical studies using IGF-1R inhibitors are reviewed here.

## ROLE OF IGF-1R IN PEDIATRIC CANCER

### EWING SARCOMA

Ewing sarcoma is the second most common bone cancer in children and adolescents. It can also occur in soft tissue. IGF-1R signaling must be intact for Ewing sarcoma cell lines to proliferate (Yee et al., 1990). Ewing sarcoma is characterized by IGF pathway activating translocations of the *EWSR1* gene. The most common translocation, found in approximately 85% of Ewing Sarcomas, is t(11;22) *EWSR1-FLI1*. The EWSR1-FLI1 gene product directly inhibits the IGFBP-3 promoter (Prieur et al., 2004). Since IGFBP-3 sequesters IGF-I from circulation (Lee and Rechler, 1996), the decreased levels of IGFBP-3 that result from the translocation may increase the bioavailability of IGF-I, thus promoting tumor growth (Kim et al., 2009). Initial preclinical testing showed that murine monoclonal antibody alphaIR3, an IGF-1R antagonist, slows *in vivo* growth of Ewing sarcoma cells in mice (Scotlandi et al., 1998). Testing by the Pediatric Preclinical Testing Program further demonstrated that growth of some Ewing sarcoma cell lines is inhibited by IGF-1R inhibition (Kolb et al., 2008, 2011). IGF-1R blockade can also be combined with other agents, specifically vincristine, doxorubicin, or imatinib for synergy (Martins et al., 2006).

### ALVEOLAR RHABDOMYOSARCOMA

Rhabdomyosarcomas are divided into two histological subtypes: embryonal, which represents about 70% of all rhabdomyosarcoma, and alveolar, which represents about 30%; pleomorphic rhabdomyosarcoma appears to be a genetically distinct sarcoma that occurs principally in adults and not in children. Although IGF activation has been implicated in driving the tumorigenicity of both rhabdomyosarcoma types, the molecular characterization of alveolar rhabdomyosarcoma (ARMS) is better understood and is therefore the focus here.

Alveolar rhabdomyosarcoma is associated with a fusion protein resulting from translocations in the *PAX3* or *PAX7* genes and *FOXO1.* The fusion protein activates the IGF-1R promoter, resulting in increased receptor expression (Ayalon et al., 2001; Xiao et al., 2002). AKT signaling secondary to up-regulation of IGF-1R has also been implicated in having an inferior outcome in ARMS patients with stage III disease, suggesting a way to risk stratify patients (Petricoin et al., 2007). As in Ewing sarcoma, rhabdomyosarcoma cell lines have demonstrated sensitivity to IGF-1R inhibition (Kolb et al., 2008, 2011).

### OSTEOSARCOMA

Osteosarcoma is the most common bone cancer in children (Mirabello et al., 2009). Osteosarcoma cell lines are dependent on IGF-1 via IGF-1R for *in vitro* growth (Kappel et al., 1994), and IGF-1R expression has been associated with poor prognosis (Wang et al., 2012). Nearly 20 years after that first observation, a mouse xenograft model using six different osteosarcoma cell lines demonstrated objective responses to R1507, a monoclonal anti-IGF1R antibody, *in vivo*. Two cell lines were resistant to R1507 (Kolb et al., 2008, 2010).

### SYNOVIAL SARCOMA

Synovial sarcoma has a peak incidence around adolescence and young adulthood (Spillane et al., 2000). It is characterized by t(X;18) translocations which increase IGF pathway mediated signaling (Sun et al., 2006). Higher IGF-1R expression has also been associated with more aggressive synovial sarcoma (Xie et al., 1999). In preclinical studies, all of 15 synovial sarcomas tested showed significant IGF-II expression and 78% showed activated IGF-1R. The IGF-1R inhibitor AEW541 had promising effects on proliferation. It also impaired cell migration (Friedrichs et al., 2008).

### WILMS TUMOR

The role of IGF-1R signaling in the pathogenesis of Wilms tumor was proposed at least as early as 1989, when an anti-IGF-1R antibody slowed tumor growth in an *in vivo* model (Gansler et al., 1989). Although Wilms tumor is generally responsive to current treatment regimens, a relatively small proportion of patients will develop recurrence (Kalapurakal et al., 2004). Increased gene copy number of IGF-1R has been associated with recurrence, and in general with worse outcomes in Wilms tumor (Natrajan et al., 2006). In spite of this evidence, little progress has been made studying the potential for IGF modulation in Wilms tumor. Initial results using Wilms tumor cell lines were promising (Houghton et al., 2010; Kolb et al., 2011), however technical difficulties growing and maintaining the cells in culture has hampered further research. Most recently, a mouse xenograft model in which cells from a Wilms tumor cell line were grown orthotopically within mouse kidney was used to show that AEW541, an IGF-1R inhibitor, reduced tumor growth (Bielen et al., 2012). It will be interesting to see if this finding can be translated to additional Wilms tumor cell lines, and in the clinic.

### NEUROBLASTOMA

Neuroblastoma represents about 10% of pediatric malignancies and is the most common cancer in the first year of life. When high risk or metastatic, survival rates are on the order of 40–50% (Maris, 2010). Like several of the other tumor types discussed here, IGF signaling was implicated in the survival of neuroblastoma cells decades ago (El-Badry et al., 1989). Neuroblastoma cell lines are sensitive to the IGF-1R inhibitor BMS-536924 (Huang et al., 2009). Additionally, IGF-1R is a major determinant of the metastatic potential of neuroblastoma. Cell lines highly expressing IGF-1R were much more likely to develop osteolytic lesions when injected into mouse tibia compared to the same cells without IGF-1R. This is thought to be secondary in part to IGF-1 chemoattraction from bone stromal cells, allowing for a microenvironment that is conducive to tumor growth (van Golen et al., 2006). More recent laboratory studies showed that the addition of temozolomide to anti-IGF-1R agents improved both *in vitro* and *in vivo* responses compared to either agent alone. Interestingly, responsiveness to anti-IGF-1R murine antibody EM164 was not related to IGF-1R expression but was correlated with decreased AKT phosphorylation after treatment (Geoerger et al., 2010). Additional preclinical studies showed both single agent anti-IGF-1R activity and additive effects when combined with more standard chemotherapies in some neuroblastoma cell lines (Wojtalla et al., 2012).

### GLIOBLASTOMA

Although usually seen in adults, glioblastoma does occur in children. Genetically, pediatric gliomas are more commonly associated with PDGFR-alpha aberrations compared to adult gliomas, which are more commonly associated with aberrations in EGFR signaling (Paugh et al., 2010). Gene amplification of IGF-1R has been shown in high grade pediatric gliomas (Bax et al., 2010; Schiffman et al., 2010). Preclinical studies combining IGF-1R inhibition with imatinib, which among other things inhibits PDGFR, showed hints of activity that are encouraging in this usually highly chemotherapy-resistant tumor type (Bielen et al., 2011).

### MEDULLOBLASTOMA

Medulloblastoma is the most common brain malignancy in children, accounting for about 25% of all pediatric intracranial cancers (Northcott et al., 2012). IGF-1R is highly expressed in a majority of medulloblastoma, with one series reporting that 80% of medulloblastomas highly express IGF-1R (Del Valle et al., 2002). There were hints of antiproliferative activity of R1507 in some medulloblastoma cell lines. Interestingly, response did not seem to be correlated with level of IGF-1R expression (Wojtalla et al., 2012).

### GASTROINTESTINAL STROMAL TUMOR

Gastrointestinal stromal tumors (GIST) occur in both children and adults. In adults, approximately 85% of GIST is at least partially driven by mutations in *KIT* or *PDGFRA*, a finding that led to the current imatinib- and sunitinib-based regimens used to treat most adult GIST (Demetri et al., 2002, 2006). On the other hand, pediatric GIST usually does not have the *KIT* and *PDGFRA* mutations that normally characterize adult GIST (Prakash et al., 2005; Janeway et al., 2007). This so-called wild type GIST is less responsive to imatinib and sunitinib (Janeway et al., 2009; Janeway and Pappo, 2012).

Insulin-like growth factor signaling dysregulation is also present in GIST from both age groups. Adult GIST is more commonly characterized by overexpression of IGF2. Pediatric GIST is more commonly associated with higher expression of IGF-1R (Agaram et al., 2008; Janeway et al., 2010; Italiano et al., 2012). The mechanism by which IGF-1R is overexpressed in these patients, and its clinical significance, remains unclear.

## MECHANISMS OF RESISTANCE

### RESISTANCE MECHANISMS AND PRECLINICAL DRUG COMBINATION STUDIES IN EWING SARCOMA

Several different resistance mechanisms have been demonstrated in Ewing sarcoma. Garofalo et al. (2012) described common pathways through which Ewing sarcoma cell lines are resistant to multiple classes of IGF-1R antagonists. In all resistant lines tested, IGF-1R was down-regulated in spite of maintenance of the ability of treatment to lead to internalization and degradation of IGF-1R after exposure to the drug, suggesting that alternative signaling cascades independent of IGF-1R drive resistance. Up-regulation of insulin receptor (IR)-A and IGF-II was seen in multiple cell lines and seems to be one alternative signaling pathway allowing for resistance (Garofalo et al., 2012). Similarly, resistant cell lines also up-regulate the MAPK/ERK pathway. MAPK signaling was recently demonstrated to be a compensatory mechanism in Ewing sarcoma cell lines after exposure to an IGF-1R specific agent, particularly through phosphorylation of AKT (Huang et al., 2011). Finally, Potratz et al. (2010) showed that BMS-536924 resistant Ewing sarcoma cells up-regulated the activity of distal IGF-1R signaling components such as mTOR and ribosomal protein S6 (RPS6). Cells that did not up-regulate these distal proteins were sensitive to the IGF1R inhibitor. siRNA knockdown of RON (MST1R, macrophage stimulating 1 receptor tyrosine kinase), restored sensitivity in BMS-536294-resistant Ewing sarcoma cell lines by decreasing RPS6 activation. In preclinical studies, at least one Ewing sarcoma cell line demonstrated enhanced response with IMC-A12 combined with rapamycin (Kolb et al., 2012).

### RESISTANCE MECHANISMS AND PRECLINICAL DRUG COMBINATION STUDIES IN ARMS

The observation of paradoxical activation of AKT with mTOR inhibitors led to the development of combination treatment using pretreatment with IGF-1R inhibitors in addition to mTOR inhibitors (Wan et al., 2007). *In vivo* studies combining IGF-1R inhibitors with rapamycin resulted in more sustained antitumor effect compared to either agent alone in a mouse xenograft model (Cao et al., 2008). In resistant ARMS cell lines, Huang et al. (2010) demonstrated different resistant mechanisms for different cell lines. In Rh41-807R cells, which are resistant to a small molecule dual-kinase inhibitor blocking both IGF-1R and IR, PDGFRA up-regulation proved to be the underlying cause of resistance. In Rh41-MAB391R cells, resistant to MAB391, an IGF-1R blocking antibody, AXL overexpression seems to be the culprit leading to resistance, although this cause and effect relationship is less clear.

IGF-1R-Her2 heterodimerization is another mechanism by which RMS cells develop resistance to IGF modulating agents. *In vitro* studies with ARMS cells demonstrated that co-treatment with NVP-AEW541, an IGF1R inhibitor, combined with lapatinib, a HER2 antagonist, reduced the phosphorylation of IGF-1R and consequently decreased IGF mediated signaling in cells resistant to IGF-1R modulators alone (Abraham et al., 2011).

### RESISTANCE MECHANISMS AND PRECLINICAL DRUG COMBINATION STUDIES IN OSTEOSARCOMA

The two cell lines that were resistant to R1507 *in vivo*, mentioned previously, both had evidence of MAPK phosphorylation through mTOR signaling, representing a possible escape mechanism in these lines after exposure to IGF-1R inhibition (Kolb et al., 2010). More recently, two osteosarcoma cell lines were among the lineages that showed enhanced activity when exposed to both IGF-1R antibody with rapamycin (Kolb et al., 2012). Ultimately, further studies are needed to assess the role of IGF modulation as a potential treatment modality for osteosarcoma.

## CLINICAL TRIALS

In clinical studies with IGF-1R antagonists alone, responses in patients were seen in some Ewing sarcoma patients (**Table 1**) and in isolated cases in patients with other cancers. Although some of these responses were quite dramatic, as a whole these results and the relatively small overall response rates are disappointing considering the plethora of promising preclinical data that preceded the early clinical trials.

**Table 1:** IGF-1R inhibitors in Ewing sarcoma (adapted from Maki, 2012).

Reference	Drug	Phase	Response (%)
Pappo etal. (2011)	R1507	II	11/115 (9.6)
Juergens etal. (2011)	Figitumumab	I–II	15/106 (14)
Malempati etal. (2012)	Cixutumumab	I–II	1/35 (2.9)
Tap etal. (2012)	Ganitumab	II	1/18 (5.6)
Tolcher etal. (2009)	Ganitumab	I+tail	2/12 (16)
Olmos etal. (2010)	Figitumumab	I-II	2/16 (12)
		TOTAL	41/312 (13)

As a result of our relatively new understanding of the role of alternative signaling pathways providing an avenue for IGF-1R antagonist resistance, canonical AKT activation has been emphasized as a possible target for clinical development of combination regimens. Clinical studies are currently underway combining IGF-1R inhibitory agents with mTOR inhibitors. Some encouraging responses were seen in these preliminary studies. In a phase I dose-expansion trial combining the IGF-1R inhibitor cixutumumab and mTOR inhibitor temsirolimus, 7 of 20 patients had either stable disease or responses by RECIST criteria (35%), and 29% had tumor regression. Interestingly, one of the six patients who had previously developed resistance to a different single agent IGF1R inhibitor had a complete response (CR) in this study (Naing et al., 2012). A second phase I study combining an IGF-1R inhibitor and mTOR inhibitor, figitumumab and everolimus, also saw cases of promising responses. This time, the most pronounced response was in a patient with solitary fibrous tumor (Quek et al., 2011).

At least one phase II study is seeking to expand on the findings of the phase I studies combining IGF-1R and mTOR targeted agents. Here, cixutumumab and temsorilimus had PFS rates of 32–42% after 12 weeks. Patients with Ewing sarcoma in the study had a response rate of 20%, with responses seen also in osteosarcoma, chondrosarcoma, and solitary fibrous tumor patients. Interestingly, in this study there was no clear correlation between biochemical markers of IGF-1R or mTOR pathway inhibition and clinical response (Schwartz et al., 2012).

Most of the studies discussed above were not targeted to the pediatric population even though in many cases adolescents and young adults were included. Seven clinical trials were identified in the clinical trials database that were designed specifically to assess pediatric cancer, including patients less than 16 years old. Of those studies identified, three are ongoing and only one is actively recruiting patients as of early 2013 (**Table 2**).

**Table 2:** Clinical trials with IGF-1R antagonists including pediatric patients.

Trial #	Drug	Phase	Sponsor/PI	Status	Publication
NCT00560144	R1507	I	Hoffmann-La Roche	Completed	
NCT00609141	Cixutumumab	I	Suman Malempati, M.D.	Completed	Malempati etal. (2012)
NCT00976508	Figitumumab + Pegvisomant	I	Pfizer	Terminated	
NCT01182883	Cixutumumab + Temsirolimus	I	Dennis D Hickstein, M.D.	Withdrawn prior to enrollment	
NCT00617890	SCH 717454	II	Merck	Active, not recruiting	
NCT00642941	R1507	II	Hoffmann-La Roche	Active, not recruiting	Pappo etal. (2011)
NCT0161479	Cixutumumab+ Temsirolimus	II	Lars M. Wagner, M. D.	recruiting	

## FUTURE DIRECTIONS

In spite of the low response rates in most trials of IGF-1R inhibitors, the dramatic images seen in the patients who did respond encourage further research in this important signaling pathway. We are only beginning to understand the mechanisms by which cancers are resistant to IGF-1R targeted agents. Clinical trials aimed at overcoming this resistance are now underway, and more will surely aid in revealing which patients may best respond to IGF-1R therapies, which may require a combination regimen, and which may not respond at all. To date, very little is known in terms of predicting who may respond. Our understanding of the potential use of IGF-1R targeted agents is less well understood in children compared to adults. Although there is a plethora of basic science data both published and forthcoming, clinical data are still lacking. IGF-1R inhibitors remain a promising focus of investigation for pediatric cancer.

## Conflict of Interest Statement

The authors declare that the research was conducted in the absence of any commercial or financial relationships that could be construed as a potential conflict of interest.
